# Outcomes of In-hospital Cardiac Arrest: Insights from a Medical Intensive Care Unit

**DOI:** 10.1371/journal.pone.0340658

**Published:** 2026-06-22

**Authors:** Karthik Kailasam, Mahmoud Alwakeel, Xiaofeng Wang, Abhijit Duggal, Sudhir Krishnan

**Affiliations:** 1 Internal Medicine, University of Arizona-College of Medicine Phoenix, Phoenix, Arizona, United States of America; 2 Critical Care Medicine, Duke university health system, Durham, North Carolina, United States of America; 3 Respiratory Institute, Cleveland Clinic Foundation, Cleveland, Ohio, United States of America; Universitatsklinikum Regensburg, GERMANY

## Abstract

**Background:**

Critically ill patients admitted to intensive care units (ICU) usually suffer from life-threatening illnesses, and many are hemodynamically unstable. The incidence of cardiac arrest in the ICU is approximately 22 per 1000 admissions, and survival to discharge after in-hospital cardiac arrest (IHCA) is approximately 14%. Variables associated with IHCA survival are poorly understood and the outcomes of cardiopulmonary resuscitation (CPR) in the ICU are poorly reported in the literature. We investigated the characteristics of IHCA and factors that are associated with poor IHCA survival.

**Methods and Findings:**

After adjusting for age, APACHE III score, and initial rhythm, every one-minute increase in CPR duration was associated with 1.161 (95% CI 1.119–1.204; p < 0.0001) odds of death during resuscitation and 1.154 (95% CI 1.059–1.258; p < 0.0001) odds of death at the time of ICU discharge. Hospital survivors had a lower APACHE III score (Mean = 88.3, SD 29.8, IQR 66–106) and acute physiology score (Mean = 75, SD 30, IQR 56–94) compared to non-survivors. Hospital survivors were also more likely than non-survivors to have a shockable rhythm at the time of arrest (20% versus 7.5%), shorter average CPR duration (5.4 minutes versus 12.8 minutes), longer length of ICU stay (14 days versus 1.8 days) and longer length of hospital stay (25 days versus 6.1 days).

**Conclusion:**

Based on our retrospective analysis, we conclude that the odds of IHCA mortality is directly proportional to the duration of CPR regardless of age, initial rhythm, and severity of underlying illness.

## Introduction

There are approximately 292,000 in-hospital cardiac arrests (IHCA) in the United States every year, with one in four survivors [1]. The factors associated with survival and favorable neurological outcomes following IHCA are poorly understood. Girotra et al showed improvements in IHCA survival to discharge from 13.7% in 2000, up to 22.3% in 2009. Among those survivors, 85% were discharged from the hospital with a favorable neurological outcome [2]. Survival following IHCA decreases with increasing age, especially among elderly patients (age > 70) [3]. Kagawa et al found IHCA patients were often older and sick with underlying co-morbid conditions [4].

Altered metabolic parameters, inadequate organ perfusion, decompensation of comorbid conditions and medical interventions are some of the variables that can precipitate a cardiopulmonary arrest in the ICU. Moskowitz et al found that delay in responding to impending respiratory failure, incorrect diagnosis, and delay in responding to clinical deterioration were some of the preventable targets for cardiac arrest in ICU [5]. Approximately 59% of all IHCA occur in the ICU, with only 14% survival during the hospitalization [6].

Variations in the reported incidence of cardiac arrest, the relevance of these findings to the specific healthcare system, and the potential for under-reporting plague our understanding of IHCA and CPR outcomes in the ICU [7]. In our retrospective observational study, we attempt to identify patient characteristics of IHCA in the ICU and determine the impact of duration of cardiopulmonary resuscitation (CPR) on survival outcomes. We hypothesize that prolonged CPR in critically ill patients admitted to the ICU is associated with higher mortality among those who survive the initial resuscitation.

## Materials and methods

### Data source and Study population

We conducted an institutional review board-approved, single-center, 5-year retrospective study of all critically ill patients > 18 years of age (N = 367) who had an in-hospital cardiac arrest (January 2014 to December 2018). Patients were admitted to the 64-bed adult medical intensive care unit at the Cleveland Clinic. The study was approved by the institutional review board of Cleveland Clinic Foundation (Study No. 19 820) on 7/23/2019. In this retrospective investigation, the ethics committee has waived the requirement for informed consent. All personal identifiers were eliminated before data analysis to safeguard patient confidentiality. The authors access to patient data was permitted within the study period (August 2019 to July 2020). All access to identifiable participant information were terminated after September 2020 and results are presented in aggregate to maintain anonymity.

Clinical data were obtained from Epic® electronic medical record. The following data points were extracted and analyzed: Demographics, Acute Physiology and Chronic Health Evaluation (APACHE) III score, Acute Physiology Score (APS), initial rhythm at the time of cardiac arrest, duration of CPR, length of hospital stay, length of ICU stay, survival after ROSC, survival at ICU discharge, survival at hospital discharge, and underlying comorbid medical conditions. Comorbid conditions included in our data collection are diabetes mellitus, chronic kidney disease on dialysis (CKD), chronic obstructive lung disease (COPD), cirrhosis, presence of immunosuppression, sepsis, and malignancy. Acute coronary syndromes leading to cardiac arrest are admitted to our dedicated cardiac ICU and hence the lack of cardiac comorbid conditions like congestive heart failure (CHF) in our study population. APACHE III scores were calculated for each patient from data collected during the first 24 hours of ICU admission.

### Statistical analysis

The Chi-square test was used to evaluate the associations between categorical variables (i.e., gender, admission source, ROSC, work shift, comorbid conditions, and condition at ICU discharge) and the duration of CPR. The Wilcoxon rank-sum test was used to examine the association of continuous variables (age, length of stay, and APACHE III score) with the duration of CPR. Study variables significant at p < .10 in the univariate analyses were explored in multivariate regression models. Variables significant at p < .05 were retained in the final regression models. A correlation analysis of the predictors was conducted to avoid multicollinearity in the model of mortality. Associations between these variables were expressed as odds ratios with 95% CIs. Statistical analyses were performed using SAS software, version 9.4 (SAS Institute). The level of statistical significance was set at p < .05 (two-tailed).

## Results

The baseline characteristics of our study population and characteristics of IHCA based on the duration of resuscitation are noted in Table 1 and Table 2 respectively. The mean age was 61 years (IQR, 51–73) of which 55% were men. The mean duration of resuscitation was 11.5 minutes (IQR, 4–17). Return of spontaneous circulation (ROSC) was obtained in 224 patients (61%) of which 82 survived to ICU discharge (23.4%) and 60 patients (16.3%) survived to hospital discharge. Prolonged CPR (>15 minutes) was associated with poor outcomes (N = 6; 5.5%).

**Table 1:** Patient characteristics.

	CPR < 5 min (N = 111)	CPR 5–10 min (N = 91)	CPR 10–15 min (N = 55)	CPR > 15 min (N = 110)	Total (N = 367)	P-value
**Mean age (SD)**	63.8 (14.9)	61.6 (18.2)	57.7 (13.7)	59.5 (15.9)	61.1 (16.0)	0.0470
**Male Sex**	62 (57.9%)	41 (48.2%)	32 (59.3%)	57 (54.8%)	192 (54.9%)	0.5050
**Mean APACHE III score (SD)**	102.2 (36.2)	104.0 (38.8)	108.0 (44.7)	92.3 (38.1)	100.6 (39.0)	0.1111
**Mean Acute Physiology score**	85.8 (35.8)	88.0 (37.2)	92.6 (42.8)	78.3 (36.6)	85.2 (37.7)	0.1813
**Diabetes Mellitus**	29 (26.9%)	30 (35.3%)	17 (31.5%)	38 (36.5%)	114 (32.5%)	0.4449
**Chronic dialysis**	17 (15.9%)	15 (17.6%)	12 (22.2%)	17 (16.3%)	61 (17.4%)	0.7707
**Cirrhosis**	13 (12.0%)	10 (11.8%)	9 (16.7%)	9 (8.7%)	41 (11.7%)	0.5244
**Severe COPD**	16 (14.4%)	20 (22.0%)	8 (14.5%)	20 (18.2%)	64 (17.4%)	0.6963
**Immunosuppression**	33 (30.6%)	25 (29.4%)	19 (35.2%)	31 (29.8%)	108 (30.8%)	0.8929
**Malignancy**	27 (24.3%)	20 (22.0%)	15 (27.3%)	28 (25.5%)	90 (24.5%)	0.8971
**Sepsis**	27 (25.2%)	25 (29.4%)	23 (42.6%)	20 (19.2%)	95 (27.1%)	0.0167

**Table 2:** Characteristics of cardiac arrests in the ICU.

	CPR < 5 min (N = 111)	CPR 5–10 min (N = 91)	CPR 10–15 min (N = 55)	CPR > 15 min (N = 110)	Total (N = 367)	P-value
**INITIAL RHYTHM**						0.9567
**Asystole**	20 (18.7%)	13 (14.8%)	12 (22.6%)	20 (19.0%)	65 (18.4%)	
**Pulseless Electrical Activity (PEA)**	77 (72.0%)	68 (77.3%)	37 (69.8%)	73 (69.5%)	255 (72.2%)	
**Pulseless Ventricular Tachycardia**	6 (5.6%)	5 (5.7%)	3 (5.7%)	9 (8.6%)	23 (6.5%)	
**Ventricular Fibrillation (VF)**	4 (3.7%)	2 (2.3%)	1 (1.9%)	3 (2.9%)	10 (2.8%)	
**Return of Spontaneous Circulation**	93 (83.8%)	74 (81.3%)	31 (56.4%)	26 (23.6%)	224 (61.0%)	<0.0001
**Survival at ICU discharge**	40 (37.0%)	23 (27.1%)	7 (13.0%)	12 (11.5%)	82 (23.4%)	0.0003
**Survival at Hospital discharge**	34 (30.6%)	16 (17.6%)	4 (7.3%)	6 (5.5%)	60 (16.3%)	<0.0001
**Median Length of hospital stay (days)**	10.8	10.8	4.1	6.6	7.9	0.0063
**Median Length of ICU stay (days)**	4.7	2.2	1.3	2.2	2.8	0.0066

### Survival based on duration of CPR

After adjusting for age, APACHE III score, and initial rhythm; every one-minute increase in CPR duration was associated with 1.161 (95% CI 1.119–1.204; p < 0.0001) odds of death during acute resuscitation and 1.154 (95% CI 1.059–1.258; p < 0.0001) odds of death at the time of ICU discharge.

**Fig 1:**
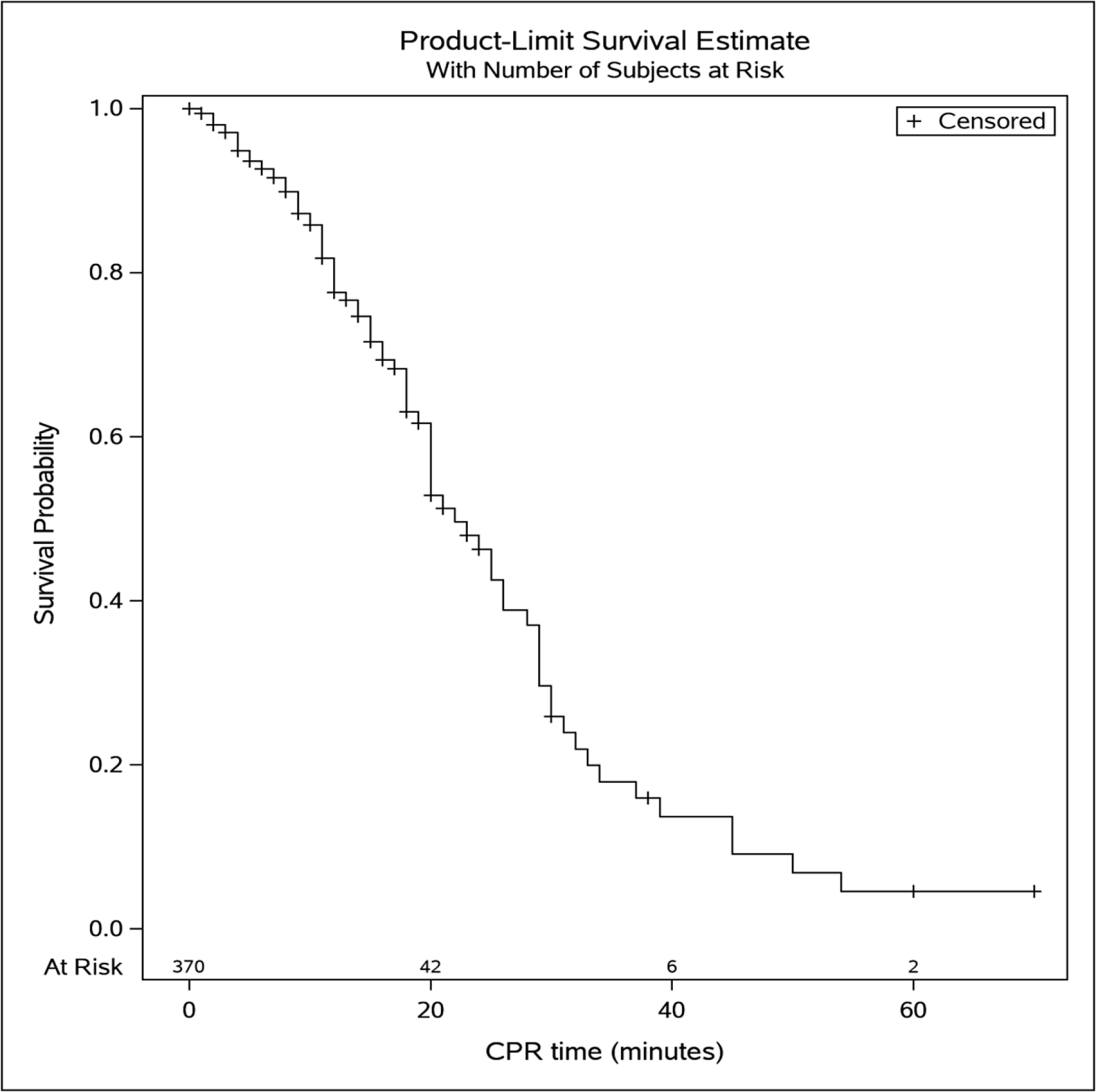
Kaplan-Meier survival curve illustrating the relationship between CPR duration (minutes) and survival probability.

**Fig 2:**
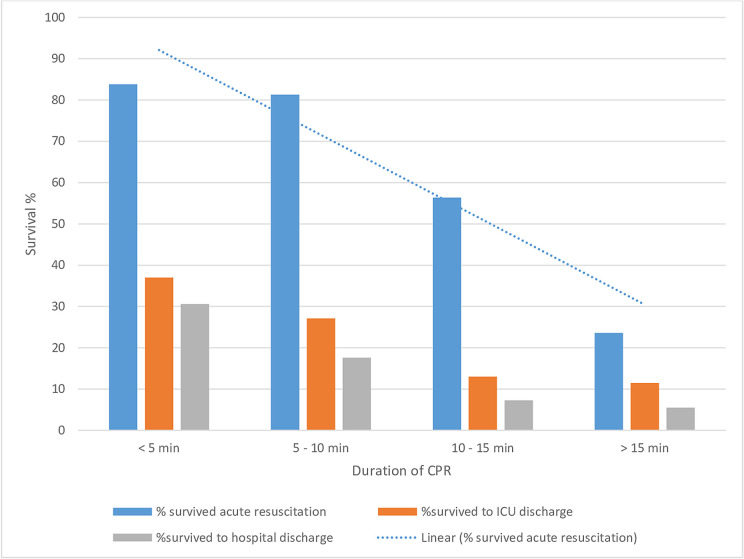
Rates of acute resuscitation survival, ICU discharge survival, and hospital discharge survival according to CPR duration. Survival rates across all three clinical endpoints demonstrate a steady decline as the duration of resuscitation increases.

The Kaplan-Meier plot illustrating survival probability relative to resuscitation duration is presented in Fig 1. Additionally, Fig 2 displays patient outcomes stratified by 5-minute increments of CPR duration.

### Hospital survival

Sixty patients survived to hospital discharge (16.3%). Hospital survivors were marginally younger (mean age of 59; SD 18) of which 55% were men. The APACHE III score (Mean = 88.3, SD 29.8, IQR 66–106) and acute physiology score (Mean = 75, SD 30, IQR 56–94) were lower compared to non-survivors. Hospital survivors were more likely than non-survivors to have a shockable rhythm at the time of arrest (20% vs 7.5%), shorter average CPR duration (5.4 minutes versus 12.8 minutes), a longer length of ICU stay (14 days versus 1.8 days) and longer length of hospital stay (25 days versus 6.1 days). Characteristic features of hospital survivors and non-survivors are listed in Table 3.

**Table 3:** Characteristics of hospital survivors compared to non-survivors.

	Hospital survivor (N = 60)	Hospital death (N = 307)
Asystole (%)	12 (20%)	56 (18.2%)
Pulseless electrical activity (%)	36 (60%)	228 (74.5%)
Pulseless ventricular tachycardia (%)	6 (10%)	18 (5.9%)
Ventricular fibrillation (%)	6 (10%)	5 (1.6%)
Mean age (SD)	59.3 (18)	61 (16)
Male (%)	33 (55%)	165 (53.7%)
Mean APACHE III (SD)	88.3 (29.8)	103 (40.6)
Mean Acute Physiology Score (SD)	75 (30)	87.1 (39.4)
Median ICU LOS in days	14	1.8
Median hospital LOS in days	25	6.1
Mean CPR duration (SD)	5.4 (4.8)	12.8 (10.6)

ROSC Achievement (Table 4): Out of 367 total patients, 224 (61%) successfully achieved Return of Spontaneous Circulation (ROSC). The duration of CPR was drastically shorter for patients who obtained ROSC compared to those who died without achieving it (Median: 5.0 minutes vs. 18.0 minutes; p < 0.0001).

**Table 4:** Outcome at ROSC.

	Died – no ROSC (N = 143)	Obtained ROSC (N = 224)	Total (N = 367)	p value
**CPR duration (minutes)**				<0.01
Mean (SD)	17.7 (10.5)	7.5 (7.3)	11.5 (10.0)	
Median	18.0	5.0	8.0	
Q1, Q3	10.0 - 25.0	3.0 - 10.0	4.0, 17.0	

ICU Survival Post-ROSC (Table 5): Among the 224 patients who initially achieved ROSC, 82 (36.6%) survived to ICU discharge. Patients who survived the ICU had significantly shorter resuscitation times than those who achieved ROSC but subsequently died in the ICU (Median: 4.0 minutes vs. 7.0 minutes; p = 0.0001).

**Table 5:** ICU survival for patients with ROSC.

	Died (N = 142)	Survived (N = 82)	Total (N = 224)	p value
**CPR duration (minutes)**				<0.01
Mean (SD)	8.5 (8.2)	5.2 (4.0)	7.4 (7.2)	
Median	7.0	4.0	5.0	
Q1, Q3	4.0 - 10.0	3.0 - 6.0	3.0 - 10.0	

## Discussion

In our study, we report ICU-IHCA survival of 16.3% at hospital discharge in comparison to previously reported 14% survival to discharge after IHCA in ICU patients over the years 2003–2010 [6]. Improved survival rate in our study population is likely related to continued advancements in resuscitation methods and clinical care over the last decade [8,9].

In our analysis, patients with CPR lasting longer than 15 minutes were relatively younger with a mean age of 59 (SD 15.9), compared to patients with CPR lasting less than 5 minutes with a mean age of 64 (SD 14.9). Khan et al reported that younger age (18–40 vs > 65 years; odds ratio [OR] 1.81; 95% CI 1.69 to 1.95; *P* < 0.001) and female sex (OR 1.05; 95% CI 1.02 to 1.09; *P* = 0.005) were associated with longer duration of resuscitation in patients who did not obtain ROSC after cardiac arrest [10]. It is assumed that this observed difference could be related to a perception among health care workers that younger patients have a higher probability of survival and merit a longer duration of resuscitation [9]. In contrast to our study results, a nationwide Swiss data analysis by Amacher et al reported women were less frequently admitted to ICUs, received fewer advanced treatments, and had a higher risk of ICU mortality when compared to men [11].

Patients with chronic illnesses such as CHF, CKD, COPD, malignancy, and cirrhosis who had IHCA were found to have significantly higher mortality in our analysis. Stapleton et al reported increased risk for death after IHCA among older adults with severe COPD (Hazard ratio 1.39; 95%CI 1.32–1.48; p < 0.001), severe CHF (HR 1.81; 95% CI 1.75–1.87; p < 0.001), severe CKD (HR 1.58; 95% CI 1.52–1.64; p < 0.001), severe cirrhosis (HR 1.69; 95% CI 1.40–2.05; p < 0.001) and malignancy (HR 1.70; 95% CI 1.58–1.84; p < 0.001) when compared to patients without those co-morbid conditions [12]. Our patients were acutely ill with a mean APACHE III score of 102.3. In our study population, 17.4% were on chronic dialysis, 32.5% had diabetes, 17.4% had severe COPD, 11.7% had cirrhosis and 30.8% were immunosuppressed (Table 1).

Furthermore, 27% of our IHCA patients had sepsis with a mortality rate of 87% at hospital discharge. This is similar to IHCA mortality in patients with severe sepsis reported by Koivikko et al [13]. Champigneulle et al reported a six-month survival rate of 14% among cancer patients admitted to the Intensive care unit (ICU) after a cardiac arrest [14]. In our cohort, 25% had an underlying malignant condition (leukemia, lymphoma, or solid tumor with metastasis) with a mortality rate of 93% at hospital discharge.

In our study, we noticed an increase in mortality proportional to the duration of resuscitation. Fernando et al in their meta-analysis of 23 cohort studies reported lower odds of survival (OR 0.12; 95% CI 0.07 to 0.19; p < 0.001) in patients with CPR longer than 15 minutes [15]. Radeschi et al studied the outcomes of IHCA in Italy and reported the median duration of CPR for survivors was 8 minutes and 26 minutes for non-survivors [16]. Kantamineni et al and Rafati et al also reported better outcomes when CPR lasted less than 10 minutes [17,18]. In our study, the mean duration of CPR was 5.2 minutes (IQR 2–6) for patients who survived to ICU discharge; and 16.4 minutes (IQR 7–24) for patients who died during acute resuscitation.

Prolonged resuscitation is associated with various traumatic and non-traumatic complications [19]. There is no clear definition for prolonged CPR as the ideal duration of resuscitation is unknown. Loisa et al reported that approximately 14% of IHCA are terminated early due to presumed futility [20]. American Heart Association’s cardiac arrest guidelines do not have any specific recommendations on termination of CPR.

Lately, clinical decision rules have been developed to assist physicians in terminating potentially futile resuscitation attempts [21']. Val Walraven et al proposed a model (the UN10 rule) for ‘futile CPR’ incorporating three intra-arrest variables i) unwitnessed arrest ii) non-shockable rhythm and iii) ROSC not obtained in 10 minutes [22,23]. Petek et al reported that among patients who meet the UN10 criteria for futile resuscitation, only 6.3% survived to discharge and 4.8% survived with favorable neurological function [23]. Our cohort had witnessed cardiac arrest in a monitored hospitalized setting, however, among non-survivors, 93% had a non-shockable rhythm and an average CPR duration of 12.8 minutes.

### Strengths and limitations

The key strengths of our study include the seminal nature of attempting to explore outcomes of IHCA in the ICU with a relatively large sample size. The study may be representative of IHCA ICU outcomes in comparable critical care setting. We acknowledge the limitations of our study. First, our retrospective study was conducted in a single large academic center involved in the care of complex patients that might not be reflective of patient demographics at other institutions. Acute coronary syndrome, a common cause for IHCA is not included in our study population since they were admitted to our dedicated cardiac ICU [24]. Hence, the lack survival outcomes based on cardiac comorbid conditions in our study population.

Another limitation of this study is the lack of information regarding post-arrest DNR status and the incidence of recurrent cardiac arrests within our cohort. Because both a change in code status to DNR and the occurrence of a subsequent arrest heavily influence overall mortality, the absence of these variables prevents us from evaluating their potential confounding effects on survival to hospital discharge

The GO-FAR score (Good outcome following resuscitation) classifies patients based on the likelihood of survival with good neurological outcomes on discharge following IHCA [21]. We were not able to incorporate the GO-FAR score in our model due to the lack of all 13 variables which are required for the calculation. We do not have data on neurological recovery after IHCA but only overall functional status at the time of discharge. Due to limitations in sample size, we were unable to perform a multivariate regression analysis to exclude all confounding factors which could influence survival outcomes.

## Conclusion

Based on our study findings, the mortality after IHCA increases with the increase in the duration of CPR regardless of age, initial rhythm, and severity of underlying illness. Further studies using clinical decision rules like GO-FAR score might assist the healthcare team in selecting appropriate patients who might benefit from prolonged resuscitation.
