# Supplementary material for: Outcomes of In-hospital Cardiac Arrest: Insights from a Medical Intensive Care Unit
Source: PLoS One. 2026 Jun 22;21(6):e0340658. doi: 10.1371/journal.pone.0340658 (PMC13286161; doi:10.1371/journal.pone.0340658)
Supplement: Attachment — Submitted filename: Response to reviewers.docx [file pone.0340658.s002.docx]

We thank the editors and reviewers for their thoughtful evaluation of our manuscript and their constructive feedback. Below, we provide point-by-point responses to the comments raised by the reviewers.

**Response to Reviewer #1**

**Reviewer #1:** *This study assessed prognostic factors associated with mortality in patients with in-hospital cardiac arrest (IHCA) and reported several significant findings. Overall, the study is interesting, and the manuscript is well written. However, I have one minor concern regarding the prevalence of do-not-resuscitate (DNR) orders among patients who achieved return of spontaneous circulation (ROSC) after IHCA, as well as the potential confounding effect of DNR status on the study outcomes.*

**Response:**

We completely agree with the reviewer that DNR status is a crucial factor that can influence post-arrest outcomes. Unfortunately, specific data regarding post-ROSC DNR orders were not captured in our record database for this cohort.

**Response to Reviewer #2**

**Reviewer #2:** *This study evaluated the association between cardiopulmonary resuscitation (CPR) duration and clinical outcomes in patients with in-hospital cardiac arrest (IHCA). The authors found that shorter CPR duration was associated with a higher survival rate.*

*In addition to CPR duration, it would be helpful if the causes of IHCA could be identified and reported, as the underlying etiology may significantly influence outcomes. Zudem, the use of further advanced interventions, such as extracorporeal membrane oxygenation (ECMO) and percutaneous coronary intervention (PCI), should be included in the analysis if these data are available.*

**Response:**

Regarding advanced interventions like ECMO and PCI, our institution operates under specific protocols where patients with primary cardiac/coronary diseases were admitted to a separate Cardiac ICU and excluded from this specific study population, resulting in lack of that data. We have clarified this structural aspect of our study population in the **Methods** section and limitations in our study population
